# A multilevel analysis to explain self-reported adverse health effects and adaptation to urban heat: a cross-sectional survey in the deprived areas of 9 Canadian cities

**DOI:** 10.1186/s12889-016-2749-y

**Published:** 2016-02-12

**Authors:** Diane Bélanger, Belkacem Abdous, Pierre Valois, Pierre Gosselin, Elhadji A. Laouan Sidi

**Affiliations:** Institut national de la recherche scientifique (INRS) Centre Eau Terre Environnement, 490, rue de la Couronne, Québec, Québec G1K 9A9 Canada; Centre de recherche du Centre hospitalier universitaire (CHU) de Québec, 2705, boulevard Laurier, Québec, Québec G1V 4G2 Canada; Université Laval, 2325 rue de l’Université, Québec, Québec G1V 0A6 Canada; Institut national de santé publique du Québec (INSPQ), 945, avenue Wolfe, Québec, Québec G1V 5B3 Canada; Ouranos, 550, Sherbrooke Ouest, Tour Ouest, 19e étage, Montréal, Québec H3A 1B9 Canada

**Keywords:** Canada, Heat, Deprivation, Health impact, Adaptation, Dwelling, Neighbourhood, Multilevel analysis, Index, Self-reported

## Abstract

**Background:**

This study identifies the characteristics and perceptions related to the individual, the dwelling and the neighbourhood of residence associated with the prevalence of self-reported adverse health impacts and an adaptation index when it is very hot and humid in summer in the most disadvantaged sectors of the nine most populous cities of Québec, Canada, in 2011.

**Methods:**

The study uses a cross-sectional design and a stratified representative sample; 3485 people (individual-level) were interviewed in their residence. They lived in 1647 buildings (building-level) in 87 most materially and socially disadvantaged census dissemination areas (DA-level). Multilevel analysis was used to perform 3-level models nested one in the other to examine individual impacts as well as the adaptation index.

**Results:**

For the prevalence of impacts, which is 46 %, the logistic model includes 13 individual-level indicators (including air conditioning and the adaptation index) and 1 building-level indicator. For the adaptation index, with values ranging from -3 to +3, the linear model has 10 individual-level indicators, 1 building-level indicator and 2 DA-level indicators. Of all these indicators, 9 were associated to the prevalence of impacts only and 8 to the adaptation index only.

**Conclusion:**

This 3-level analysis shows the differential importance of the characteristics of residents, buildings and their surroundings on self-reported adverse health impacts and on adaptation (other than air conditioning) under hot and humid summer conditions. It also identifies indicators specific to impacts or adaptation. People with negative health impacts from heat rely more on adaptation strategies while low physical activity and good dwelling/building insulation lead to lower adaptation. Better neighbourhood walkability favors adaptations other than air conditioning. Thus, adaptation to heat in these neighbourhoods seems reactive rather than preventive. These first multi-level insights pave the way for the development of a theoretical framework of the process from heat exposure to impacts and adaptation for research, surveillance and public health interventions at all relevant levels.

**Electronic supplementary material:**

The online version of this article (doi:10.1186/s12889-016-2749-y) contains supplementary material, which is available to authorized users.

## Background

Health impacts attributed to periods of high summer temperatures are undeniable in all latitudes [1]. To date, several scientific publications have reported on emergency room visits and hospitalizations, mainly by assessing medical consultation records. The risk factors usually refer to individual characteristics, possibly due to the type of data available in health system administrative records. Neighbourhood [2, 3], building or dwelling [4, 5] characteristics as well as coping behaviours [6–10] are considered less frequently, despite their close connection with vulnerability to heat [11].

Our study exploits the perceptual approach to estimating the adverse health impacts of heat exposure as used or recommended elsewhere [9, 12–15]. This approach has the significant advantage of considering the wide range of factors potentially having a negative effect on health but which are difficult to measure directly [12, 16] and which have to be validated [17–19]. A similar phenomenon has been observed from studies using self-reported health behaviour [20, 21]. The perceptual approach has also been used for the evaluation of environmental exposure [22]. In this regard perception of room temperature is a proximal variable of thermal comfort associated with various health problems [12, 17]. This method has recently been described as an alternative to traditional techniques for measuring ambient temperature [23].

The perceptual approach is therefore a very promising technique for adding value to health and housing studies [23]. Its use for measuring the health impacts of heat and its determinants is highly relevant from a public health perspective, particularly given that periods of high summer heat will intensify in the near future [24] and that these events are already affecting the residents of the most disadvantaged neighbourhoods in major cities [2, 3, 25] struggling with the urban heat island effect [26, 27]. These materially and socially disadvantaged census dissemination areas (DAs) in Québec are located within the province’s large urban centers [28, 29] and are characterised by a lack of green space and an abundance of asphalt [3].

Our study pertains to these geographic pockets of urban poverty localised in the hottest spaces or neighbourhoods. We studied the most disadvantaged DAs in the nine cities of Québec with a population of at least 100 000 in 2010, which constituted 47 % of the total population [30]. In this paper we aim to explain (1) self-reported adverse health impacts and (2) self-reported adaptations other than use of air conditioning at home during very hot and humid summer episodes using a multilevel approach. Dwelling DAs by way of neighbourhoods (DA-level), occupied buildings (building-level), and individuals (individual-level) represent for the analysis three-tiers or levels nested one inside the other. This multilevel approach has been used successfully in the field of health [31, 32] but to our knowledge has not yet been applied to heat-health related problems.

## Methods

A cross-sectional design with a stratified sample was chosen for this study. A protocol for the study (including a questionnaire) received approval by the research ethics committee of the *Centre hospitalier universitaire de Québec* prior to commencement of field work.

### Sample

A two-step selection technique adapted from Vallée et al. [33] was used to identify representative samples in each of the nine cities. The number of highly disadvantaged DAs per city was identified based on a widely used Canadian deprivation index [34]. Each such DA included minimally one public low-rental housing building. This first step also identified the total number of households to be included in the survey per city. The second step consisted of randomly selecting households to be approached. One adult per household was interviewed.

A total of 3485 individuals living in 1647 buildings and 87 DAs were interviewed. While the overall response rate was 19 %; another statistic is the response rate by question, which was much higher at 95 % or more for more than 90 % of questions asked (for more details, see [35, 36].

### Data collection and measures

#### Data collection

Questionnaire-based interviews were carried out by 21 interviewers who had each received two days of training. The face-to-face interviews took place in the participant’s home. The development of the draft questionnaire was inspired by relevant population survey tools such as the Statistics Canada’s 2007 questionnaire for the Canadian Community Health Survey and was pre-tested and adjusted prior to data collection. Participants received $10 as payment for their time. For more information see [35, 36].

#### Dependent variables

The first of two dependent variables was based on a proxy variable of participant response to a question about whether “their physical health was negatively affected during very hot and very humid summer conditions”. This variable, referred to as ‘self-reported adverse health impacts’, reflects the self-reported state of overall health in the context of summer heat exposure. A separate and similar question was administered pertaining to their self-reported state of “mental health” in the same context. The preamble and questions were adapted from the Canadian Community Health Surveys going back to 2001 [37]. Both of these key questions were grouped and a binary variable was used to summarize responses. Participants reporting their physical and/or mental health as moderately or greatly adversely affected by very hot and humid weather were considered as the risk group (vs. slightly or not at all).

Second, we constructed an individual adaptation index to very hot and humid summer conditions using Multiple Correspondence Analysis [38, 39]. The heat adaptation index covers 14 easy-to-use and energy-efficient adaptations for reducing the perception of heat (cooling off) or seeking protection from the sun, regardless of location (indoors/outdoors/at home or other). These adaptations are: when the sun is shining, (1) wearing a head covering, (2) closing curtains to keep the home cool; to cool off, (3) taking showers or baths more often than usual, (4) sponging the face and neck, (5) eating iced foods, (6) swimming in a public pool, lake or river, (7) frequenting places other than home for air conditioning, (8) drinking tap water as a main drink, (9) using the balcony and (10) the yard in the evening; to reduce heat sources in the home, (11) switching off the computer when not in use, (12) decreasing dryer and (13) oven use; finally, (14) adopting preventive behaviour according to weather information transmitted through the media or on the Internet (binary variables: 0 = never/rarely, 1 = occasionally/often/always). Seventy-five percent of the total inertia on dimension 1 is explained by these 14 variables (dimension 2: 5 %). Index values range from about –3 to +3 and are almost normally distributed in the case of Dimension 1 more technical details on MCA methods and the proposed index might be found in reference [40] which explains the development of the index.

#### Independent variables

The choice of independent variables was based on the scientific literature on human health and oppressive heat [41, 42]. In the current study, they reflect the three levels nested one within the other and considered within the multilevel analysis. The individual-level indicators concern heat exposure in the neighbourhood of residence, the building and the dwelling, air conditioning, index adaptation and city of residence as classified according to the average temperature over the last 30 years [43] (Additional file 1: Table S1). They also correspond to the states or conditions that increase heat sensitivity and to some indicators related to the access to treatment. For the building-level indicators, the averages of some individual-level variables for each building sampled were used in the absence of census data on buildings. Finally, the DA-level variables were evaluated using averages of some individual-level variables and with data available by DA at the *Institut national de santé publique du Québec*, including an indicator of intra-urban heat islands [44] and an index walkability [45].

### Analysis

F-test, t and Chi-square tests together with classical bivariate analyses were performed. In terms of software, survey procedures such as proc surveylogistic in SAS 9.3 were utilised. To make them more concise, bivariate analyses are presented only for the variables used also in multivariate model.

In the present multivariate analysis, the study of the self-reported adverse health impacts of heat is different from previous papers from the same dataset as we used a multilevel approach rather than generalized estimating equations (GEE) methods [35, 36]. As reported by Chaix and Chauvin and contrary to GEE, multilevel models have the specific objective to explain intergroup variation by higher level variables (here building and DA levels); the contribution of these covariables is thus presented here to explain the self-reported adverse health effects while only individual-level covariables were used in previous GEE analyses. We also added here several individual-level covariables related to heat adaptation (other than air conditioning at home) which were not present in previous analyses [35, 36]. Otherwise, the only previous article on the individual adaptation index to very hot and humid summer conditions [40] concerned its development and criterion-related validity. The present analysis thus concerns the explanation of the index in a multivariate model within a multilevel approach, including individual-level, building-level and 6 DA-level covariables.

More precisely, for the multivariate analysis, we used MLwiN [46, 47] to fit the three-level model (individual-level nested in building-level nested in DA-level). A logistic regression model served to explain the self-reported adverse health impacts (binary variable), while a linear regression model was utilised to explain the heat adaptation index (continuous variable). For both regressions, an ascending regression procedure enabled the authors to identify the main independent variables associated with the dependent variables. This means that we evaluated first if the building-level, then the DA-level, were statistically significant when added to the null-model. Secondly, we added in an ascending regression procedure the individual-level covariables (when statistically significant in bivariate analyses). Finally, the building-level and DA-level covariables contribution to the model were estimated.

The models were evaluated for the influence of the season in which the interview took place. We make sure in this article, by means of tetrachoric (binary variables) or polychoric (variables with ≥ 3 strata) correlation coefficients, that no independent variables presented (taken two at a time) were strongly correlated (r ≥ 0.6). No statistically significant cross-level interactions were observed (*p* < 0.05). Consequently no interaction terms were used. Moreover, to obtain more interpretable estimates, some of the individual-level variables were centered when the model included both the variable of individual-level and the average of this variable at building-level or DA-level [48]. Hence, the part attributed to the building mean (or DA) was substracted to estimate only the individuals contribution [49]. These centered variables are noted in Tables 1 and 2. Also, the models that examine a covariable’s influence at two levels are known as contextual or compositional models (see [50] for more details).

**Table 1:** 3-level multivariate logistic regression model of the self-reported adverse health impacts when it is very hot and humid in summer: fixed part

Fixed part	M_0_^a^	M_00_^b^	M_000_^c^	M_000:1_^d^	M_000:12_^e^
	β (ES)^f,g^	β (ES)^f,g^	β (ES)^f,g^	β (ES)^f,g^	β (ES)^f,g^
Constant	−0,103 (0,034)****	−0,135 (0,038)****	−0,120 (0,046)***	−0,571 (0,193)**	−0,678 (0,213)***
ADDITION OF INDIVIDUAL-LEVEL COVARIABLES					
Self-reported diagnoses of chronic diseases:					
≥2 diagnoses (vs none)				1.010 (0.110)****	1.014 (0.110)****
1 diagnosis (vs none)				0.325 (0.105)***	0.327 (0.105)***
Long-term leave for illness or disability (vs no)				0.825 (0.130)***	0.837 (0.131)***
Health problems due to air quality within the dwelling in the opinion of the respondents (vs no)				0.737 (0.164)****	0.740 (0.164)****
Female gender (vs no)				0.455 (0.086)****	0.454 (0.086)****
Rather or extremely stressed most of the time (vs no)				0.377 (0.096)****	0.375 (0.096)****
Air conditioning at home (vs no)				0.356 (0.082)****	0.352 (0.082)****
Neighbourhood perceived as fairly or heavily polluted due to the density of urban traffic (vs no)				0.329 (0.096)****	0.325 (0.096)****
Adaptation index (other than with air conditioning in the dwelling) when it is very hot and humid in summer (continuous)				−0.310 (0.047)****	−0.309 (0.047)****
Perceived need for more infrastructure or services in dwelling neighbourhood to adapt better when it is very hot and humid in summer:					
Yes, in urban planning (vs no)				0.265 (0.133)**	0.261 (0.133)**
Yes, in other areas such as public transport (vs no)				0.084 (0.092)	0.087 (0.092)
No physical activity in the past 3 months (vs yes)				0.249 (0.093)**	0.248 (0.093)**
Age					
18–44 (vs ≥ 65 years)				0.016 (0.130)	0.014 (0.129)
45–64 (vs ≥ 65 years)				0.222 (0.114)*	0.226 (0.114)*
≥2 caregivers in the last year living < 80 km from the dwelling but not in the same neighbourhood (vs < 2)				−0.217 (0.086)**	−0.220 (0.086)**
Satisfaction with the indoor temperature of dwelling in summer:					
Not centered (continuous)				−0.421 (0.041)**	
Centered on the building average (continuous)					−0.475 (0.059)**
Addition of building-level covariables					
By building, average satisfaction with the indoor temperature of dwelling (continuous)					−0.375 (0.055)**

^a^M_0_ = 1-level null model (individuals, I). ^b^M_00_ = 2-levels null model (I + buildings, B). ^c^M_000_ = 3-level null model (I + B + DA). ^d^M_000:1_ = 3-level model with covariables of individual-level. ^e^M_000:12_ = 3-level model with covariables of individual-level and building-level. ^f^β (ES): estimated β coefficient (standard error). ^g^Values *p*: ****: *p* ≤0.0001; ***: *p* ≤0.001; **: *p* ≤0.01; *: *p* ≤0.05

**Table 2:** 3-level multivariate linear regression model of the adaptation index when it is very hot and humid in summer: fixed part

Fixed part	M_0_^a^	M_00_^b^	M_000_^c^	M_000:1_^d^	M_000:12_^e^	M_000:123_^f^
	Β (SE)^g,h^	Β (SE)^g,h^	Β (SE)^g,h^	Β (SE)^g,h^	Β (SE)^g,h^	Β (SE)^g,h^
Constant	−0.026 (0.017)	−0.153 (0.021)****	−0.140 (0.027)****	0.727 (0.088)****	0.724 (0.094)****	0.957 (0.124)****
Addition of individual-level covariables						
Age						
18–44 (vs ≥ 65 years)				−0.566 (0.047)****	−0.565 (0.047)****	−0.572 (0.047)****
45–64 (vs ≥ 65 years)				−0.344 (0.041)****	−0.344 (0.041)****	−0.344 (0.041)****
Perceived need of more infrastructure and services in the neighbourhood of residence to adapt better when it is very hot and humid in summer:						
Yes, in urban planning (vs no)				−0.303 (0.036)****	−0.303 (0.036)****	−0.301 (0.036)****
Yes, in other areas such as public transport (vs no)				−0.151 (0.052)**	−0.150 (0.052)**	−0.150 (0.051)**
Self-reported adverse health impacts when it is very hot and humid in summer				−0.281 (0.034)****	−0.281 (0.034)****	−0.285 (0.034)****
Face to face with friends a few times a month or more (vs. no)				−0.284 (0.062)****	−0.284 (0.062)****	−0.282 (0.062)****
≥2 caregivers in the last year living in the same neighbourhood but not in the same dwelling (vs <2)				−0.209 (0.039)****	−0.209 (0.039)****	−0.207 (0.039)****
≥2 caregivers in the last year living <80 km from the dwelling but not in the same neighbourhood (vs <2)				−0.186 (0.034)****	−0.186 (0.034)****	−0.181 (0.034)****
Automobile as the primary mode of transportation for local travel within the past year (vs. no)				−0.100 (0.034)**	−0.100 (0.034)**	−0.097 (0.034)**
No physical activity in the last 3 months (vs yes)				0.332 (0.036)****	0.332 (0.036)****	0.334 (0.036)****
≥1 functional disability:						
Often (vs. never)				0.223 (0.048)****	0.223 (0.048)****	0.221 (0.048)****
Sometimes (vs. never)				0.128 (0.050)**	0.127 (0.050)**	0.125 (0.050)**
Satisfaction with quality of dwelling’s thermal insulation in summer:						
Not centered (continuous)				0.081 (0.016)****		
Centered on the building average (continuous)					0.080 (0.023)****	0.079 (0.023)****
Addition of building-level covariables						
By building, average satisfaction with quality of dwelling’s thermal insulation in summer (continuous)					0.081 (0.022)****	0.082 (0.022)****
Addition of da-level covariables						
By DA, average duration of residence in the same dwelling (continuous)						−0.028 (0.010)**
By DA, walkability index (continuous)						−0.013 (0.007)*

^a^M_0_ = 1-level null model (individuals, I). ^b^M_00_ = 2-levels null model (I + buildings, B). ^c^M_000_ = 3-level null model (I + B + DA). ^d^M_000:1_ = 3-level model with covariables of individual-level. ^e^M_000:12_ = 3-level model with covariables of individual-level and building-level. ^f^M_000:123 _= 3-level model with covariables of individual-level, building-level and DA-level. ^g^β (ES): estimated β coefficient (standard error). ^h^ *p* values: ****: *p* ≤0.0001; ***: *p* ≤0.001; **: *p* ≤0.01; *: *p* ≤0.05

For the linear regression model, the residual variance (variance not explained by the model) in the heat adaptation index has been decomposed into three components: the “within buildings between individuals variance”, the “within DA between buildings variance” and the “between DA variance”. We used the Likelihood ratio test to compare nested models and estimated the percentage of variation found at each level by the variance partition coefficients (VPC) [48]. For the logistic regression model, we did not consider partitioning variances for the perceived adverse health impacts, since a simple VPC is not available for binary variables. Variances and residual variance percentages for health impacts to heat, however, were reported by level to give an idea of the contribution of the addition of a model with regard to the previous model. Moreover, to assess if the response prevalence exhibits more or less variation than would be expected under a binomial distribution, we used the approach of multiplicative over-dispersion by adding a multiplicative scale factor (α) to the variance of the binary response (α = 1, then the prevalence is binomially distributed; α > 1 or α < 1, over-dispersion or under-dispersion respectively) [48].

## Results

### Explanation of self-reported adverse health impacts when it is very hot and humid in summer

As already reported [35, 36], the prevalence of self-reported adverse health impacts during very hot and humid summer conditions was 46.0 % (confidence interval, CI: 44.2–47.8) for the 3485 respondents living in the very disadvantaged dissemination areas of the nine most populated cities within Québec.

The random part of the logistic regression model (based on the extra-binomial distribution, since α < 1) shows that the residual variances at the building-level and DA-level decrease significantly as the model evolves (Additional file 2: Table S2). At the building-level, residual variance decreases by 33 % with the addition of the DA-level (M_000_) to the building-level null model (M_00_), by 51 % with the addition of individual-level covariables (M_000:1_) to M_000_, and by 17 % with the addition of a building-level covariable (M_000:12_) to M_000:1_. At the DA-level, residual variance decreases by 48 % from M_000_ to M_000:1_ and then stabilizes. Therefore, the use of a 3-level model appears justified.

The 3-level model (M_000:12_) is adjusted for thirteen individual-level covariables (bivariate analyses presented in the Additional file 3: Table S3) and a building-level covariable. It is not influenced by the season in which the interview took place (*p* = 0.0580), however, nor by the presence of intra-urban heat islands (66 % of visited buildings were located in a heat island and 32 % within 50 metres of one, *p* = 0.5380), and none of the DA-level covariables were statistically significant.

Of the fourteen covariables that significantly explain (*p* < 0.05) the prevalence of impacts (M_000:12_, Table 1), eleven are risk indicators and three are prevention indicators.

The eleven impact-risk indicators are individual-level. They include: self-reported chronic diseases, especially if there is more than one; long-term medical leave or disability; health problems due to the quality of the air inside the dwelling in the opinion of the respondents; perceived stress that is high or very high in most cases; a neighbourhood perceived as somewhat or very polluted due to urban traffic density; access to air conditioning in the dwelling; higher self-reported heat adaptation according to index (this results in a negative coefficient β in the model); the perception that urban development infrastructure must be added in the dwelling neighbourhood in order to improve adaptation to heat; as well as not practicing physical activities in the past three months, being female and between 45 and 64 years old.

The three impact-prevention indicators include: support in the past year by at least two people living within a radius of 80 km from the place of residence but not in the same neighbourhood; and two covariables for temperature satisfaction inside the dwelling in summer, one being individual-level (correlation with air conditioning at home, *r* = −.04) and the other being building-level. According to the latter, individuals who are most satisfied with the temperature inside their dwelling in summer (coefficients reduced by −0.475 for each additional satisfaction point; so, the odds of adverse health impacts is reduced by 37.8 % for each additional satisfaction point) and the buildings in which households are on average more satisfied (coefficients reduced by −0.375 for each addition of a satisfaction point; so, the odds of adverse health impacts is reduced by 31.3 % for each additional satisfaction point) are at lower odds of self-reported adverse health impacts to heat.

### Explanation of adaptation index when it is very hot and humid in summer

Of the 3485 respondents, 16.6 % (CI 15.3–18.0) were very adaptive, according to the adaptation index (values ≤ −1), 16.7 % (CI 15.3–18.0) were hardly adaptive at all (values ≥ 1), while 66.7 % (CI 65.0–68.4) were between these two poles.

The random part of the linear regression model shows that the 3-level null model is statistically significant (Additional file 4: Table S4). Overall, it also shows that the building-level and DA-level residual variances decrease significantly as the model evolves (see relative changes) and that its adjustment for individual and contextual covariables is significant. Moreover, the 3-step null model explains 83, 14 and 2 % of the total residual variance of the adaptation index to the individual-level, building-level and DA-level, respectively. Residents’ adaptation indices for the same building (and the same DA) are therefore slightly more correlated than if they lived in different buildings.

The 3-level model thus proves to be relevant, but the aggregation rate is low. It is adjusted for ten individual-level covariables (bivariate analyses presented in the Additional file 5: Table S5), one building-level and two DA-level covariables. This model, however, is not influenced by air conditioning at home (*p* = 0.6748) nor by the season in which the interview took place (*p* = 0.0797). Of the thirteen covariables that significantly explain (*p* < 0.05) the adaptation index, four characterize a weaker tendency to adopt behaviours measured by the index (this translates into β positive coefficients in the model), and nine provide incentive to be more adaptive (negative β; Table 2).

The four covariables characterizing a weaker self-reported adaptation according to the index include: not practising physical activity in the past three months, the fact of suffering from at least one functional disability (such as difficulty walking), especially if it is often, and two covariables on satisfaction with the quality of the thermal insulation of the dwelling in summer, one of which is individual-level (correlation with air conditioning at home, *r* = −.04) and the other building-level. According to the latter, the people most satisfied with the thermal-insulation quality of their dwelling in summer (an increase of 0.79 in the index for each additional point of satisfaction) and the buildings in which households on average are more satisfied (an increase of 0.82 in the index for each additional point of average satisfaction) adopt fewer behaviours measured by the index for adapting to the heat.

Of the nine covariables that encourage more adaptive behaviour according to the index, seven are individual-level and two are DA-level. The first are: being younger than 65 years old, in particular 18 to 44; perceiving the need for more urbanistic infrastructure or services to improve adaptation to heat in the residence’s neighbourhood; feeling the adverse impacts on their health when it is very hot and humid in summer; having met with friends at least once a month and having been supported by at least two persons residing in the same neighbourhood (not the same dwelling) or less than 80 km from the dwelling but not in the same neighbourhood in the last year; and primarily using a car for local travel. The final two reveal that, the lower the residential mobility in a DA (decrease in the index by −0.028 for each additional year to the average duration of residency in the same dwelling) and the better the potential for walking (decrease in the index by −0.013 for each additional walkability point), the more the people who live there adopt behaviours measured by the index of adaptation.

### Summary of fixed effects

Among all covariables associated with one dependent variable or another, nine explain only the impact prevalence and eight explain only the adaptation index (Additional file 6: Table S6).

## Discussion

In this study, multilevel analyses made it possible to process building-level and DA-level variance as a source of information [51], in contrast to other approaches used to analyze the same dataset [35, 36].

The results of random parts of the analyses show that a 3-level model supports the identification of indicators associated with self-reported adverse health impacts and the adaptation index when it is very hot and humid in summer. Therefore, they emphasize the need to develop public health interventions aimed at both residents of very disadvantaged DAs, property managers in these sectors and municipal authorities in large urban centres already struggling with the urban heat island.

The importance of the role of buildings and the environment as risk (or prevention) factors of self-reported health impacts and adaptation to heat is strengthened by the contribution of four building-level and DA-level covariables. However, this number of four variables remains well below the number of individual-level covariables, despite important efforts to measure various aspects of the context (see Additional file 1: Table S1). This observation has been reported in the literature and some explanations have been given on this subject [52–54], including the fact that census data do not characterize neighbourhoods in detail and that a DA is an administrative geographical unit and not a neighbourhood as perceived by respondents.

The effect of buildings and the environment on self-reported adverse health impacts and on the adaptation index could also be covered by some covariables. We cannot make any conclusions regarding this aspect due to the cross-sectional design of our study. Also in the absence of a proven theoretical framework, because although some interesting avenues have been proposed [41, 55, 56], none have been validated. The establishment of such a framework would be very useful for research and public health surveillance, in particular for clarifying the kind of links between covariables; in our study, most are related only to the prevalence of self-reported adverse health impacts or to the adaptation index.

Specifically, in this study, the prevalence of self-reported negative health impacts when it is very hot and humid in summer is 46 %; although high, this could correspond in reality to the highly disadvantaged DAs of the most populous cities in Québec, as discussed elsewhere [35, 36].

As already reported in the literature [41, 57–59], poor physical or mental health is a powerful indicator of risk to heat. Similarly, residential neighbourhoods considered somewhat or very polluted due to high urban traffic density are in all likelihood more paved and therefore hotter. Such highly paved and thus more impermeable neighbourhoods contribute to the negative health impacts of heat [2, 3, 60, 61] and result in the deployment of various adaptations [9, 10].

In addition, hormonal differences may explain the difference in self-reported adverse health impacts by gender [62, 63] for women aged 45 to 64 in particular (menopause period) [35]. Regardless of gender, the 45–64 age group, however, remain at higher odds of impacts according to our results. This is contrary to what is generally reported in this area of research [42] but plausible in very poor populations such as ours. In fact, the proportion of 45–64 years old having more chronic health problems increases according to a decrease in family income [64], while seniors having survived the same conditions would in all likelihood have been in better health. Another explanation for the increased odds of heat impacts for the 45–64 group would be greater exposure, mainly because of the requirement to leave home for family or work reasons [35] compared to older people, who are more confined [10]. Clarifying this issue would be important for public health because those 65 and over are generally the target group for national heatwave plans.

Physical inactivity, a risk factor for several non-communicable diseases [57], is associated with a higher prevalence of self-reported adverse health impacts. The average level of physical activity declines with age; the result is a downward trend in fitness [64, 65]. It is known that poor physical fitness leads to a low cardiovascular reserve and a low tolerance to humid heat [66]. However, physical inactivity was not associated with self-reported adverse health impacts in multivariate models in our previous articles [35, 36]. It is thus possible that this multilevel analysis highlights a potential contribution of deprived neighbourhoods to physical inactivity [67, 68]. Further studies are needed on this.

In our study, physical inactivity, like functional limitations, is also associated with a lower self-reported adaptation to heat, according to the index. More specifically, these two conditions characterize very poor populations [64, 65], such as our sample drawn from DAs that are farther (in time and distance) from cool greenspaces than more affluent DAs [69].

Improving the urban development of the poorest sectors of large urban centres would then be more beneficial for countering the health impacts of heat and facilitating adaptation. Over a third of respondents in our study also expressed this need. In addition, this initiative would have many co-benefits [55, 57]. For example, according to our results, DAs that offered better walkability were on average more adaptive according to the index, which could mean a reduction in the use of air conditioning in the home [70], which contributes to the outside heat and indirectly by exacerbating the air pollution in some cases [57]. Similarly, better equipped DAs could reduce residential mobility and improve levels of well-being in neighbourhoods [71, 72] while encouraging adaptation by means other than air conditioning in the dwelling. These recent hypotheses, however, remain to be confirmed.

As already published [7, 42], but not in our previous multivariate analyses [35, 36], social support is associated with health impacts from heat with this multilevel approach. According to our results in the present article, the network that helps prevent impacts would be less diverse and more likely to be family than the network that promotes adaptation in a way other than air conditioning in the home. Further studies are needed in this respect, as networks with strong links could potentially exacerbate rather than reduce vulnerability to the effects of heat in the elderly, according to some authors [73].

The relationship between satisfaction with the indoor temperature of the dwelling in summer and the decrease in the prevalence of self-reported adverse health impacts, as well as the relationship between satisfaction with insulation quality and reduced adoption of behaviours measured by index of adaptation, clearly highlights the contribution of the building occupied in reducing exposure to heat, beyond the individual sensitivities. The fact that each of these relationships is observed for both the dwelling (individual-level) and the building (building-level) reinforces the importance of the use of these perceptions for public health surveillance. Especially in highly disadvantaged areas of major urban centres, where there are old buildings whose insulation meets less efficient standards than today’s, unless upgrading has been done since they were built [36].

Last, in this study the variables were self-reported which is generally considered easy to implement and cost-effective in several situations. As reported elsewhere [35], the validity of self-reported versus medical-based diagnoses and behaviours has been well established over time, several countries and data collection methods, especially as a tool for predicting future risks and as an epidemiologic survey tool for prevention and public health actions. Perceived health is thus a reliable and valid subjective measurement of the overall state of health and is widely used by statistical and health agencies throughout the world. This self-reporting approach for evaluating environmental exposures is also documented if not as well assessed. Moreover, the objective measures are far from perfect, as already mentioned [12, 17]. From a public health perspective, it remains important to validate these results in other contexts.

## Limitations of the study

While the study’s response rate was low, the rate by question was very high. The latter is a another measure of the survey’s response rate [74]. To our knowledge there have been no comparable studies published, particularly with respect to certain key aspects of the sample selection criteria (very disadvantaged DAs in Canada) and therefore evaluating the response rate in light of comparative work is not possible. However, the response rate reported for a qualitative study undertaken in two of the same sample sites as our study [75] and thus reflecting similar community characteristics such as large urban centres, multiethnic environments, high density, etc., indicates that our response rate could represent what can realistically be obtained in the very disadvantaged DAs of Québec’s largest cities. It is also in line with recent trends in response rates in North America [76].

In following our ethics protocol, no information was collected from people refusing participation. In response to this limitation, some statistical comparisons with census data available by DA were undertaken at the *Institut national de santé publique du Québec*. In fact, there are only two minor discrepancies between our sample and census data. Indeed, our sample is older (mean age 53 vs 47) and richer (mean income 30 k vs 24 k). This might be explained by the oversampling of public housing in the study (half of sample). That said, these differences should not influence health impacts and adaptation behaviours because all the proposed models have been adjusted for age, while income was not a significant variable (see Tables 1 and 2). Therefore we can state that despite the low classic response rate, the study samples acceptably represented the populations of the DAs included in the study as well as those of the very disadvantaged DAs in Québec’s nine largest cities, due to the sampling plan that was adopted in the study (for more details, see [35, 36]. It follows that reliable generalizations of this study’s results to deprived neighbourhoods with similar characteristics can be made.

## Conclusion

This research, conducted in the most disadvantaged DAs in the most populous cities in Québec in 2011, is highly innovative in many ways. In particular, it shows that the use of a multi-level approach helps better identify the differential contribution of the characteristics of residents, buildings and their surroundings on self-reported adverse health impacts and adaptation (other than air conditioning in the home) when it is very hot and humid during the summer. On the other hand, both at the individual-level and building-level, satisfaction levels with indoor temperature in summer would be good indicators of exposure to heat at home, while the quality of satisfaction levels with their thermal insulation (in summer) are good indicators of the ability to counter exposure to heat. Considered more broadly, it appears that some indicators are more specific to impacts while others primarily characterize adaptation. Finally, people who feel negative health impacts from heat rely more on various mechanical and non-mechanical adaptation strategies as other residents of very disadvantaged DAs. Thus, adaptation to heat might be deployed in response to the impacts of heat, rather than to prevent them.

Further studies are needed to test these hypotheses. In particular, it would prove important to develop a theoretical framework ranging from exposure to self-reported adverse health impacts under very hot and humid summer conditions, including adaptation to heat as well as the characteristics and perceptions related to the life environment. Filling this gap will make a major contribution to research because the establishment of a theoretical framework helps clarify the type of links that exist between the indicators associated with the issues, define how to measure and improve monitoring, and improve surveillance and the public health response. It would also help the eventual development of national databases on housing and the neighbourhood. The establishment of observatories [77] in public health and climate change would support the achievement of these goals.

## Additional files

Additional file 1:
**Independent variables by level.** (DOCX 19 kb) Additional file 2:
**3-level multivariate logistic regression model of the self-reported adverse health impacts when it is very hot and humid in the summer: random part.** (DOCX 16 kb) Additional file 3:
**Individual-level covariables associated with the prevalence of self-reported adverse health impacts when it is very hot and humid in summer: bivariate analysis.** (DOCX 26 kb) Additional file 4:
**3-level multivariate linear regression model of the adaptation index when it is very hot and humid in summer: random part.** (DOCX 19 kb) Additional file 5:
**Individual-level covariables associated with the adaptation index when it is very hot and humid in summer.** (DOCX 20 kb) Additional file 6:
**Synthesis of the results regarding the fixed effects of the Impact and Index models.** (DOCX 18 kb)
