# Supplementary material for: A multilevel analysis to explain self-reported adverse health effects and adaptation to urban heat: a cross-sectional survey in the deprived areas of 9 Canadian cities
Source: BMC Public Health. 2016 Feb 12;16:144. doi: 10.1186/s12889-016-2749-y (PMC4751716; doi:10.1186/s12889-016-2749-y)
Supplement: Additional file 1: — Independent variables by level. (DOCX 19 kb) [file 12889_2016_2749_MOESM1_ESM.docx]

**Supplementary Table 1:**

**Independent variables by level**

| **LEVEL-1: Individuals** |
| --- |
| 1. Sociodemographic: gender, age, education level |
| 1. Socioeconomic (12 months): household income (before deductions), principal occupation |
| 1. Sociocultural: place of birth, major languages for conversation, reading, length of residence in Canada and citizenship status if immigrants |
| 1. Household: type of household, number of children |
| 1. Social contacts (12 months): frequency of contact by telephone and face to face with family, friends, neighbours, participation as a member of an organization/non-profit association |
| 1. Social support (12 months): number of caregivers living in the same neighbourhood but not in the same dwelling, number of caregivers living < 80 km but not in the same neighbourhood |
| 1. Lifestyle: physical activities, body mass index (3 months), smoking, every day or almost in dwelling, drinking alcohol, and main mode of transport (12 months) |
| 1. Clinical health: Number of self-reported diagnoses of chronic illnesses |
| 1. Perceived state of health: perceived health status, quality of life, high stress in most cases, health problems due to air quality within dwelling |
| 1. Disabilities: ≥ 1 functional disability, ≥ 1 physical/mental disability, need help getting around neighbourhood^A^ |
| 1. Care/health services (12 months): ≥ 1 medical consultation ≥ 1 night in a hospital/nursing home/convalescent home, number of hours per week of care and services at home |
| 1. Accommodation: duration of residence, number of persons per room, per bedroom, floor occupied, rooms under roof, dwelling infected by animals or harmful insects, need for maintenance or repairs, satisfaction with dwelling characteristics (internal T° in summer, etc.) |
| 1. Building: type of building (≥ 5 floors, etc.), parking, elevator, type and colour of cladding, satisfaction with building characteristics (security, etc.) |
| 1. Neighbourhood^A^: duration of residence, opinions on various issues in neighbourhood (air pollution from road traffic density, etc.), sense of belonging, security |
| 1. City: hometown classified according to the average temperature of the last 30 years |
| 1. Adaptation when it is very hot and humid in summer: access to air conditioning in the dwelling^C^, perceived need for more services/infrastructure to adapt better in the neighbourhood of residence, adaptation index other than with air-conditioning (to explain the impacts) |
| 1. Self-reported adverse health impacts when it is very hot and humid in summer (to explain the adaptation index) |
| **LEVEL-2: Buildings** |
| - Level 1 variable averages (points 12 and 13) |
| **LEVEL-3: DA** |
| - Level 1 variable averages (points 12 to 14) and DA indicators provided by the Institut national de santé publique du Québec: intra-urban heat island indicator , walkability index, normalized vegetation index, distance between residence postal code and nearest recreational infrastructure, distance between residence postal code and nearest park or green space, 2006 census data (population density, % of dwellings in need of major repairs, % of ≥ 65%, % of women, % of people living alone) |

**^A^** Neighbourhood: all that lies within a 15-20 minute walk from home, when walking at a normal and regular pace.

**^B^** Of the respondents who have access to air conditioning at home, 90% used it during the day and in the evening when it is very hot and humid in summer; the others also used it at night.
