# Supplementary material for: A multilevel analysis to explain self-reported adverse health effects and adaptation to urban heat: a cross-sectional survey in the deprived areas of 9 Canadian cities
Source: BMC Public Health. 2016 Feb 12;16:144. doi: 10.1186/s12889-016-2749-y (PMC4751716; doi:10.1186/s12889-016-2749-y)
Supplement: Additional file 2: — 3-level multivariate logistic regression model of the self-reported adverse health impacts when it is very hot and humid in the summer: random part. (DOCX 16 kb) [file 12889_2016_2749_MOESM2_ESM.docx]

**Supplementary Table 2:**

**3-level multivariate logistic regression model of the self-reported adverse health impacts when it is very hot and humid in the summer: random part**

| **RANDOM PART** | **M_00_^A^** | **M_000_^B^** | **(M_00_-M_000_)**  **/M_00_^F^** | **M_000:1_^C^** | **(M_000_-M_000:1_)**  **/M_000_^F^** | **M_000:12_^D^** | **(M_000:1_-M_000:12_)**  **/M_000:1_^F^** |
| --- | --- | --- | --- | --- | --- | --- | --- |
|  | **σ^2^ (SE)^E^** | **σ^2^ (SE)^E^** |  | **σ^2^ (SE)^E^** |  | **σ^2^ (SE)^E^** |  |
| **Levels (L)** |  |  |  |  |  |  |  |
| L3 - DAs |  | 0.058 (0.027) |  | 0.030 (0.027) | .48 | 0.031 (0.028) | -.03 |
| L2 - buildings | 0.158 (0.057) | 0.106 (0.056) | 0.33 | 0.052 (0.062) | .51 | 0.043 (0.061) | .17 |
|  |  |  |  |  |  |  |  |
| **α (CI)^G^** | 0.96  (0.91-1.01) | 0.96  (0.91-1.01) |  | 0.985  (0.93-1.04) |  | 0.987  (0.93-1.04) |  |
|  |  |  |  |  |  |  |  |
| **Units**^H^ |  |  |  |  |  |  |  |
| L3 - AD |  | 87 |  | 86 |  | 86 |  |
| L2 - buildings | 1 646 | 1 646 |  | 1 488 |  | 1 488 |  |
| L1- Individuals | 3 484 | 3 484 |  | 3 092 |  | 3 092 |  |

^A^M_00_ = 2-level null model (I+buildings, B). ^B^M_000_ = 3-level null model (I+B+DA). ^C^M_000:1_ = 3-level model with covariables of individual-level. ^D^M_000:12_ = 3-level model with covariables of individual-level and building-level. ^E^σ^2^(SE) = variance (standard error).

^F^ These proportions represent unexplained (or residual) variance reduction of the prevalence of health impacts when moving from one model to another. ^G^Extra-binomial parameter (confidence interval). ^H^ Observed differences between models are due to missing data for one variable or more.
