# Supplementary material for: A multilevel analysis to explain self-reported adverse health effects and adaptation to urban heat: a cross-sectional survey in the deprived areas of 9 Canadian cities
Source: BMC Public Health. 2016 Feb 12;16:144. doi: 10.1186/s12889-016-2749-y (PMC4751716; doi:10.1186/s12889-016-2749-y)
Supplement: Additional file 3: — Individual-level covariables associated with the prevalence of self-reported adverse health impacts when it is very hot and humid in summer: bivariate analysis. (DOCX 26 kb) [file 12889_2016_2749_MOESM3_ESM.docx]

**Supplementary Table 3:**

**Individual-level covariables associated with the prevalence of self-reported adverse health impacts when it is very hot and humid in summer: bivariate analysis**

| **Covariables** | **% ^A^ (CI) ^B^** | **P ^C^ (CI) ^B^** |
| --- | --- | --- |
| **Exposure to heat** |  |  |
| Neighbourhood perceived as fairly or heavily polluted due to the density of urban traffic |  |  |
| Yes | 50.3 (40.8-51.5) | 52.3 (49.8-54.8) |
| No | 49.8 (48.0-51.5) | 40.0 (37.5-42.5) |
| Satisfaction with indoor temperature of dwelling in summer (not centered) |  |  |
| Completely dissatisfied | 21.9 (20.0–23.9) | 72.2 (67.8–76.5) |
| Somewhat dissatisfied or satisfied | 56.8 (54.4–59.1) | 41.2 (38.1–44.2) |
| Completely satisfied | 21.3 (19.4–23.3) | 32.5 (27.7–37.3) |
| **Existing state of health** |  |  |
| Self-reported chronic disease diagnoses |  |  |
| Yes, ≥ 2 diagnoses | 31.7 (30.1-33.3) | 64.1 (61.1-67.0) |
| Yes, 1 diagnosis | 24.6 (23.0-26.1) | 44.3 (40.8-47.9) |
| No diagnosis | 43.7 (42.0-45.5) | 33.9 (31.3-36.5) |
| Long-term leave for illness or disability |  |  |
| Yes | 16.1 (14.8-17.4) | 72.2 (68.2-76.2) |
| No | 83.9 (82.6-85.3) | 40.9 (39.1-42.9) |
| Health problems due to air quality within the dwelling, in the opinion of the respondents |  |  |
| Yes | 8.5 (7.5–9.5) | 72.6 (67.0–78.2) |
| No | 91.5 (90.5–92.5) | 43.5 (41.7–45.4) |
| Rather or extremely stressed most of the time |  |  |
| Yes | 24.8 (23.3–26.3) | 57.8 (54.4–61.2) |
| No | 75.2 (23.3-26.4) | 42.2 (40.1-44.2) |
| **Lifestyle** |  |  |
| Practises physical activity, past 3 months |  |  |
| No, never | 32.1 (30.4-33.7) | 51.1 (48.0-54.2) |
| Yes, < 1 time/day | 35.1 (33.4-36.8) | 45.0 (42.0-48.0) |
| Yes, ≥ 1 time/day | 32.9 (31.2-34.6) | 42.2 (39.1-45.4) |
| **Social support** |  |  |
| ≥ 2 caregivers having supported respondent in past year and living < 80 km from dwelling but not in same neighbourhood |  |  |
| No caregiver | 53.8 (51.5-56.1) | 49.6 (46.3-52.9) |
| 1 caregiver | 16.6 (14.8-18.3) | 45.1 (39.3-50.9) |
| 2 caregivers | 13.1 (11.5-14.7) | 43.6 (37.2-50.1) |
| ≥ 3 caregivers | 16.5 (14.8-18.3) | 39.4 (33.6-45.3) |
| **Adaptation when it is very hot and humid in summer** |  |  |
| Air conditioning at home |  |  |
| Yes^D^ | 49.5 (47.7-51.2) | 50.9 (48.4-53.4) |
| No | 50.5 (48.8-52.3) | 41.3 (38.9-43.8) |
| Adaptation index |  |  |
| ≤ -1 | 16.6 (15.3-18.0) | 57.8 (53.5-62.1) |
| < 1, goal > -1 | 66.7 (65.0-68.4) | 46.3 (44.1-48.4) |
| ≥ 1 | 16.7 (15.3-18.0) | 33.4 (29.1-37.6) |
| Perceived need of more infrastructure or services in the neighbourhood of residence to adapt better when it is very hot and humid in summer |  |  |
| Yes, in urban planning | 38.1 (36.3-39.8) | 58.3 (53.2-63.5) |
| Yes, in other areas such as public transport | 12.1 (10.9-13.3) | 48.5 (45.6-51.3) |
| No | 49.8 (48.1-51.6) | 42.0 (39.4-44.6) |
| **Sociodemographic attributes** |  |  |
| Gender |  |  |
| Female | 54.2 (52.5-56.1) | 51.9 (49.7-54.1) |
| Male | 45.8 (44.0-47.6) | 39.1 (36.3-41.9) |
| Age |  |  |
| 18-44 years | 31.0 (28.9-33.1) | 41.0 (36.9-45.0) |
| 45-64 years | 39.8 (37.5-42.0) | 52.9 (49.2-56.6) |
| ≥ 65 years | 29.3 (27.2-31.4) | 42.1 (37.8-46.5) |

**^A^** %: weighted frequencies in percentages. Percentages have been rounded to one decimal place. **^B^** IC: confidence interval

**^C^** P: prevalence of self-reported adverse health impacts when it is very hot and humid in summer. For each variable, the difference between groups was < 0.0001, except for “≥ 2 caregivers having supported respondent in past year and living < 80 km from dwelling but not in same neighbourhood” (p=0.0005). **^D^** Of these respondents: (a) 80% had a window air conditioner (mobile: 10% had a wall or central a/c: 10%); (b) more than 90% cooled their dwelling during the day and in the evening with a/c when it is very hot and humid in summer; the other respondents used it at night as well.
