# Supplementary material for: A multilevel analysis to explain self-reported adverse health effects and adaptation to urban heat: a cross-sectional survey in the deprived areas of 9 Canadian cities
Source: BMC Public Health. 2016 Feb 12;16:144. doi: 10.1186/s12889-016-2749-y (PMC4751716; doi:10.1186/s12889-016-2749-y)
Supplement: Additional file 4: — 3-level multivariate linear regression model of the adaptation index when it is very hot and humid in summer: random part. (DOCX 19 kb) [file 12889_2016_2749_MOESM4_ESM.docx]

**Supplementary table 4:**

**3-level multivariate linear regression model of the adaptation index when it is very hot and humid in summer: random part**

| **Random part** | **_M0_^A^** | **_M00_^B^** | **(M0-M_00_)**  **/M_0_^H^** | **M_000_^C^** | **(M_00_-M_000_)**  **/M_00_^H^** | **M_000:1_^D^** | **(M_000_-M_000:1_)**  **/M_000_** | **M_000:12_^E^** | **(M_000:1_-M_000:12_)**  **/M_000:1_^H^** | **M_000:123_^F^** | **(M_000:12_-M_000:123_)**  **/M_000:12_^H^** |
| --- | --- | --- | --- | --- | --- | --- | --- | --- | --- | --- | --- |
|  | **σ^2^ (ES)^G^** | **σ^2^ (ES)^G^** |  | **σ^2^ (ES)^G^** |  | **σ^2^ (ES)^G^** |  | **σ^2^ (ES)^G^** |  | **σ^2^ (ES)^G^** |  |
| **Levels (L)** |  |  |  |  |  |  |  |  |  |  |  |
| L-DA |  |  |  | 0.022 (0.009) |  | 0.010 (0.005) | 0.55 | 0.010 (0.005) | 0.00 | 0.008 (0.005) | 0.20 |
| L-buildings |  | 0.158 (0.023) |  | 0.138 (0.023) | 0.13 | 0.022 (0.012) | 0.84 | 0.022 (0.012) | 0.00 | 0.020 (0.011) | 0.09 |
| L-individuals | 0.974 (0.023) | 0.806 (0.024) | 0.17 | 0.806 (0.024) | 0.00 | 0.713 (0.021) | 0.12 | 0.713 (0.021) | 0.00 | 0.713 (0.021) | 0.00 |
| **Total** | 0.974 | 0.964 |  | 0.966 |  | 0.745 |  | 0.745 |  | 0.741 |  |
|  |  |  |  |  |  |  |  |  |  |  |  |
| **-2**log likelihood*** | 9 797.84 | 9 610.13 |  | 9 598.39 |  | 7 604.56 |  | 7 604.51 |  | 7 595.20 |  |
| **RV^I^ p values** |  | 0.000 |  | 0.001 |  | 0.000 |  | 0.808 |  | 0.010 |  |
|  |  |  |  |  |  |  |  |  |  |  |  |
| **Units^J^** |  |  |  |  |  |  |  |  |  |  |  |
| L-DA |  |  |  | 87 |  | 87 |  | 87 |  | 87 |  |
| L-buildings |  | 1 647 |  | 1 647 |  | 1 438 |  | 1 437 |  | 1 437 |  |
| L-individuals | 3 485 | 3 485 |  | 3 485 |  | 2 998 |  | 2 998 |  | 2 998 |  |

^A^M_0_ = 1-level null model (individuals, I). ^B^M_00_ = 2-level null model (I+buildings, B). ^C^M_000_ = 3-level null model (I+B+DA). ^D^M_000:1_ = 3-level model with covariables of individual-level. ^E^M_000:12_ = 3-level model with individual-level and building-level covariables. **^F^**M_000:123_ = 3-level model with individual-level, building-level and DA-level covariables. **^G^** σ^2^(ES) = variance (standard error). ^H^ These proportions represent unexplained (or residual) variance reduction of the adaptation index of health impacts when moving from one model to another. **^I^** RV: likelihood ratio test. **^J^** The differences observed between the models are due to missing data for one or more covariables.
