# Supplementary material for: A multilevel analysis to explain self-reported adverse health effects and adaptation to urban heat: a cross-sectional survey in the deprived areas of 9 Canadian cities
Source: BMC Public Health. 2016 Feb 12;16:144. doi: 10.1186/s12889-016-2749-y (PMC4751716; doi:10.1186/s12889-016-2749-y)
Supplement: Additional file 5: — Individual-level covariables associated with the adaptation index when it is very hot and humid in summer. (DOCX 20 kb) [file 12889_2016_2749_MOESM5_ESM.docx]

**Supplementary Table 5:**

**Individual-level covariables associated with the adaptation index when it is very hot and humid in summer**

| **Bivariate analyses** | | | | |
| --- | --- | --- | --- | --- |
| **Variables** | **%^A^ (IC)^B^** | **% according to adaptation index^C^ (IC)^B^** | | |
|  |  | **Low** | **Medium** | **High** |
| **Exposure to heat** |  |  |  |  |
| Satisfaction with quality of dwelling’s thermal insulation in summer |  |  |  |  |
| Completely dissatisfied | 22.5 (21.1–24.0) | 11.2 (8.0-14.3) | 69.1 (64.6-73.7) | 19.7 (15.8-23.5) |
| Somewhat dissatisfied or satisfied | 58.7 (56.9-60.4) | 16.0 (13.7-18.4) | 66.5 (63.6-69.5) | 17.5 (15.1-19.8) |
| Completely satisfied | 18.8 (17.4–20.2) | 25.6 (20.6-30.6) | 63.5 (58.0-69.0) | 10.9 (7.3-14.5) |
| **Disability** |  |  |  |  |
| ≥ 1 functional disability |  |  |  |  |
| Never | 71.1 (69.5-72.7) | 14.2 (12.2-16.3) | 67.0 (64.3-69.6) | 18.9 (16.7-21.0) |
| Sometimes | 12.7 (11.5-13.9) | 19.7 (14.6-24.8) | 64.5 (58.2-70.8) | 15.7 (10.7-20.8) |
| Often | 16.1 (14.9-17.5) | 25.1 (20.1-30.2) | 67.4 (61.9-72.8) | 7.5 (4.5-10.5) |
| **Lifestyle** |  |  |  |  |
| Practises physical activities (3 months) |  |  |  |  |
| Yes | 32.0 (30.4-33.7) | 12.1 (10.1-14.0) | 68.2 (65.5-70.9) | 19.8 (17.4-22.1) |
| No | 68.0 (66.3-69.6) | 26.4 (22.7-30.1) | 63.6 (59.6-67.5) | 10.0 (7.8-19.3) |
| Automobile main mode of local transport (12 months) |  |  |  |  |
| Yes | 42.3 (40.6-44.0) | 16.0 (13.2-18.7) | 63.4 (59.9-66.9) | 20.7 (17.7-23.6) |
| No, public transit | 57.7 (56.0-59.4) | 17.1 (13.2-18.7) | 69.2 (66.3-72.1) | 13.7 (11.6-15.9) |
| **Support and social contacts in the past year** |  |  |  |  |
| ≥ 2 caregivers living in the same neighbourhood (not in same dwelling) |  |  |  |  |
| ≥ 2 caregivers | 41.6 (39.8-43.4) | 14.0 (11.4-16.6) | 66.9 (63.4-70.3) | 19.2 (16.3-22.0) |
| 1 caregiver | 35.8 (34.1-37.5) | 16.6 (13.6-19.7) | 66.3 (62.6-70.1) | 17.0 (14.0-20.0) |
| No caregiver | 22.6 (21.1-24.1) | 20.9 (16.7-25.2) | 67.1 (62.3-72.0) | - 1. (8.5-15.3) |
| ≥ 2 caregivers living < 80 km from dwelling (not in same neighbourhood) |  |  |  |  |
| ≥ 2 caregivers | 29.6 (28.0-31.3) | 11.7 (8.8-14.7) | 65.8 (61.6-70.0) | 22.5 (18.8-26.1) |
| 1 caregiver | 16.6 (14.8-18.3) | 16.2 (11.7-20.6) | 66.7 (61.1-72.3) | 17.2 (12.7-21.6) |
| No caregiver | 53.8 (51.5-56.1) | 19.5 (16.9-22.2) | 67.7 (64.6-70.7) | 12.8 (10.6-14.9) |
| Face to face with friends a few times a month or more |  |  |  |  |
| Yes | 7.0 (6.1-6.9) | 15.6 (13.8-17.5) | 66.8 (64.4-69.1) | 17.6 (15.8-19.5) |
| No | 93.0 (92.1-94.0) | 27.1 (19.1-35.0) | 68.0 (59.7-76.3) | 4.9 (1.2-8.6)^D^ |
| **Adaptation when it is very hot and humid in summer** |  |  |  |  |
| Perceived need for infrastructure or services to adapt better to the neighbourhood of residence |  |  |  |  |
| Yes, in urban planning | 38.1 (36.3-39.8) | 9.7 (7.4-12.0) | 67.3 (63.8-70.9) | 23.0 (19.8-26.2) |
| Yes, in other areas such as public transit | 12.1 (10.9-13.3) | 12.6 (8.3-16.8) | 70.5 (64.2-76.7) | 17.0 (11.6-22.3) |
| No | 49.8 (48.1-51.6) | 22.7 (19.8-25.6) | 65.4 (62.2-68.7) | 11.9 (9.7-14.1) |
| **Health impacts when it is very hot and humid in summer** |  |  |  |  |
| Self-reported impacts |  |  |  |  |
| Yes | 46.0 (44.2-47.8) | 12.1 (9.8-14.4) | 67.0 (63.8-70.3) | 20.9 (18.0-23.7) |
| No | 54.0 (52.2-55.7) | 20.6 (17.9-23.2) | 66.4 (63.4-69.5) | 13.0 (19.9-15.1) |
| **Sociodemographic attributes** |  |  |  |  |
| Age |  |  |  |  |
| 18-44 years | 31.0 (28.9-33.1) | 7.1 (4.7-9.4) | 65.7 (61.8-69.7) | 27.2 (23.5-30.9) |
| 45-64 years | 39.8 (37.5-42.0) | 15.0 (12.3-17.7) | 68.8 (62.3-72.3) | 16.2 (13.4-19.1) |
| ≥ 65 years | 29.3 (27.2-31.4) | 29.1 (25.1-33.1) | 64.9 (60.7-69.1) | 6.0 (3.9-8.1) |

**^A^** %: weighted frequencies in percentages. Percentages have been rounded to one decimal place. **^B^** IC: confidence ^C^ In this table, the adaptation index values were divided into three groups: high for values ≤ -1, medium for values < 1, but > -1, and low for values ≥ 1. For each covariable, the differences between the groups have a value of p < 0.0001.
