# Supplementary material for: A multilevel analysis to explain self-reported adverse health effects and adaptation to urban heat: a cross-sectional survey in the deprived areas of 9 Canadian cities
Source: BMC Public Health. 2016 Feb 12;16:144. doi: 10.1186/s12889-016-2749-y (PMC4751716; doi:10.1186/s12889-016-2749-y)
Supplement: Additional file 6: — Synthesis of the results regarding the fixed effects of the Impact and Index models. (DOCX 18 kb) [file 12889_2016_2749_MOESM6_ESM.docx]

**Supplementary Table 6:**

**Synthesis of the results regarding the fixed effects of the Impact and Index models**

| **Covariables** | **Impacts^A^** | **Index^A^** |
| --- | --- | --- |
| **INDIVIDUAL-LEVEL** |  |  |
| **Exposure to heat** |  |  |
| Neighbourhood perceived as fairly or heavily polluted due to density of urban traffic | ↑ | NA |
| Indoor dwelling temperature deemed satisfactory in summer by respondents | ↓ | NA |
| Quality of dwelling thermal insulation in summer deemed satisfactory by respondents | NA | ↓ |
| **Existing state of health** |  |  |
| ≥ 2 self-reported diagnoses of chronic diseases | ↑ | NA |
| Long-term leave for illness or disability | ↑ | NA |
| Health problems due to air quality within dwelling in the opinion of respondents | ↑ | NA |
| Rather or extremely stressed most of the time | ↑ | NA |
| **Disability** |  |  |
| ≥ 1 functional disability, mainly if they manifest themselves often | NA | ↓ |
| **Lifestyle** |  |  |
| Automobile as the primary mode of transportation for local travel in the past year | NA | ↑ |
| No physical activity within the past 3 months | ↑ | ↓ |
| **Support and social contact in the past year** |  |  |
| ≥ 2 caregivers living in the same neighbourhood (not in the same dwelling) | NA | ↑ |
| ≥ 2 caregivers living < 80 km from the dwelling (not in the same neighbourhood) | ↓ | ↑ |
| Face to face with friends a few times a month or more | NA | ↑ |
| **Adaptation when it is very hot and humid in summer** |  |  |
| Air conditioning in the dwelling | ↑ | NA |
| High adaptation index | ↑ |  |
| Perceived need for more urban infrastructure to adapt better to the neighbourhood | ↑ | ↑ |
| Perceived need for more infrastructure or services other than urban development (e.g., public transit) to better adapt to the neighbourhood of residence | NA | ↑ |
| **Health impacts when it is very hot and humid in summer** |  |  |
| Self-reported adverse health impacts |  | ↑ |
| **Sociodemographic attributes** |  |  |
| Female gender | ↑ | NA |
| Age Mainly 45-64 | ↑ | NA |
| Age Mainly 18-44 | NA | ↑ |
| **BUILDING-LEVEL** |  |  |
| **Exposure to heat** |  |  |
| By building, average satisfaction of indoor temperature of dwelling in summer | ↓ | NA |
| By building, average satisfaction with quality of thermal insulation of dwelling in summer | NA | ↓ |
| **DA-LEVEL** |  |  |
| **Adaptation to heat** |  |  |
| By DA, better walkability index | NA | ↑ |
| By DA, greater average duration of residence in the same dwelling | NA | ↑ |
| **Total covariables associated with a single dependent^B^ variable** | 9 | 8 |

**^A^** ↑ : risk indicators associated with increased prevalence of self-reported adverse health impacts or negatively affecting adaptation according to index; ↓: indicators associated with decreased prevalence of impacts or facilitating adaptation according to index; NA: not applicable. **^B^** These totals do not include self-reported adverse health impacts and the index of adaptation, as they could only be associated with a single dependent variable or with the four covariables associated with impacts and the index.
